# Improving Physician-patient and Physician-nurse Communication and Overall Satisfaction Rates: A Quality Improvement Project

**DOI:** 10.7759/cureus.7776

**Published:** 2020-04-22

**Authors:** Asif Hitawala, Monica Flores, Mohammad Alomari, Sany Kumar, Vinay Padbidri, Sujit Muthukuru, Shafia Rahman, Ahmed Alomari, Shrouq Khazaaleh, K V Gopalakrishna, Madonna Michael

**Affiliations:** 1 Internal Medicine, Cleveland Clinic - Fairview Hospital, Cleveland, USA; 2 Internal Medicine, Cleveland Clinic Foundation, Cleveland, USA; 3 Internal Medicine, Kettering Medical Center, Dayton, USA; 4 Hematology and Medical Oncology, Albert Einstein College of Medicine, New York, USA; 5 Internal Medicine, The Hashemite University, Zarqa, JOR

**Keywords:** quality improvement, satisfaction, physician-nurse, physician-patient, communication

## Abstract

Introduction

Communication between healthcare providers and patients is a key component associated with the quality of healthcare and patient satisfaction. Often, simple communication skills may be insufficient to sustain a successful provider-patient relationship. The aim of this project was to assess and improve patient and nurse satisfaction with physicians via improvement in physician-patient and physician-nurse communication to a level greater than 90%.

Methods

Initial surveys were given to the patients and nurses on admission to the regular nursing floor to assess current satisfaction rates. Afterward, visual handouts were given that provided details about the current medical team members and the role of each team member. which were updated daily along with the medical plan. Surveys were then handed out to the patients and their nurses at the time of discharge. All surveys were conducted anonymously.

Results

A total of 26 surveys (n = 13 patients, n = 13 nurses) were collected and analyzed for a preliminary assessment. Surveys concluded that 68.8% of patients were satisfied with the patient-provider communication; similarly, 74.4% of the nurses were satisfied with the nurse-provider communication. In the next six weeks, visual handouts were implemented. During this period, surveys involving a total of 40 patients and 40 nurses were collected. The results after the intervention revealed that 93.3% of patients were satisfied with the patient-provider communication, and 94.7% of nurses were satisfied with the nurse-provider communication. Post-intervention, the Hospital Consumer Assessment of Healthcare Providers and Systems (HCAHPS) displayed an improvement in physician communication, reaching the expected goal of 84.4%.

Conclusion

Ineffective communication often goes undetected in many healthcare settings, causing serious effects on the health and safety of patients, and may ultimately jeopardize overall satisfaction. Literature has shown a positive correlation between patient satisfaction and improved clinical outcomes. Using visual aids and updating medical care plans on a daily basis are simple yet effective tools to improve communication. Written materials should be created in a patient-friendly manner to enhance communication, clarity, and understanding.

## Introduction

A doctor’s communication and interpersonal skills include the ability to gather information in order to facilitate accurate diagnosis, counsel appropriately, give therapeutic instructions, and establish caring relationships with patients [1-2]. These are part of core clinical skills in the practice of medicine and are essential to achieve the best outcomes and patient satisfaction [1,3]. Basic communication skills alone are not sufficient to create and maintain a successful physician-patient relationship, as it consists of shared perceptions and feelings regarding the nature of the problems, treatment goals, and psychosocial support [2,4]. Studies on doctor-patient communication have shown discontent even when many doctors have felt their communication to be good or excellent [5]. Research on physician-nurse collaboration has yielded similar results: physicians viewed physician-nurse collaboration as less important but rated their own quality higher than that of the nurses [6].

Solutions for Value Enhancement (SolVE) cohort is a continuous 12-week quality improvement training program sponsored by graduate medical education of the Cleveland Clinic in order to train caregivers on how to successfully plan, implement, obtain, and analyze data and then apply quality improvement initiatives. Since nurse-physician and patient-physician communication may be subpar and have been shown to affect patient outcomes, our team decided to conduct a quality improvement project in order to assess the current patient and nurse satisfaction rates regarding communication with physicians and find ways to improve them.

## Materials and methods

The project was carried out at the Cleveland Clinic-Fairview Hospital. A pre-intervention survey was sent out to a random group of patients who were admitted under the hospitalist medicine team. Another survey was sent out to their nurses.

**Figure 1:**
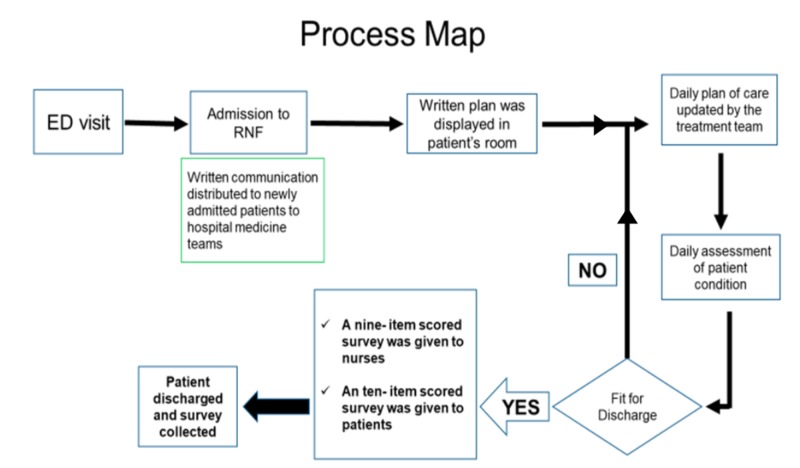
Flow diagram of the project ED: emergency department; RNF: regular nursing floor

After initial surveys were completed, visual handouts were distributed to the patients during bedside rounds with the intent to serve as a visual aid tool to communicate and update the medical plan. The handouts included details regarding the hospitalist team, contact pager, and photos with the name and role of each team member (attending and resident) followed by a chart that can be updated daily along with the medical plan. These handouts were placed in the patients’ rooms, to be visualized by the patients, patients’ families, and their nurses. They were updated on a daily basis after completing bedside rounds until discharge. At the time of discharge, a satisfaction survey was sent to the patients and their nurses (Figure 1).

Patients with a length of stay of fewer than 48 hours or those who were unwilling or unable to participate in the surveys were excluded. If any patient was willing and able to participate but his/her nurse was not, the patient was still included in the study and vice versa. All pre- and post-intervention surveys were conducted anonymously. The patient surveys included 10 questions, to be scored from 1-10 (Table 1). The nurse surveys included nine questions, to be scored from 1-10 (Table 2).

**Table 1:** Patient survey questionnaire Patients were asked to provide a rating based on each question, with 1 being least likely/helpful and 10 being most likely/helpful

Question	Score (1-10)
Did this written method help you know which physician is in charge of your overall care?	
Did this help you and your family understand your treatment plan every day?	
Did this help you ask the right questions to your physician?	
Did this help you to be more involved in the decisions about your treatment?	
Did this make it easier for you and your family in making treatment choices?	
Did this help you know your test results more clearly?	
Did this help you clarify with your nurse whatever you didn’t understand from your physician?	
Did this method help you discuss your anxieties and fears regarding your medical illness with your physician?	
Did this written method increase your confidence in your treatment team?	
Would you prefer the written method in addition to the verbal method of communication by your treatment team?	
Total	

**Table 2:** Nurse survey questionnaire Nurses were asked to provide a rating from 1 to 10, with 1 being least favorable and 10 being most favorable

Question	Score (1-10)
How easy was it to identify the primary physician in charge of the patient?	
How easy was it to reach the primary physician in charge of the patient?	
How well did you understand the goals of care for the patient?	
What was the effect on communication with patients and families with the intervention?	
What was the effect on communication between different healthcare providers with the intervention?	
Did the intervention improve overall patient care?	
How easy is it to identify primary physicians in emergency situations?	
How satisfied are you with the intervention?	
How likely are you to recommend this in the future?	
Total	

The final score was then calculated out of a total of 100 and 90 for the patient and nurse surveys, respectively. The final data was then compiled and analyzed.

## Results

A total of 26 surveys (n = 13 patients, n = 13 nurses) were collected and analyzed for a preliminary assessment. Surveys concluded that 68.8% of the patients were currently satisfied with the patient-provider communication; similarly, 74.4% of the nurses were satisfied with the nurse-provider communication.

**Figure 2:**
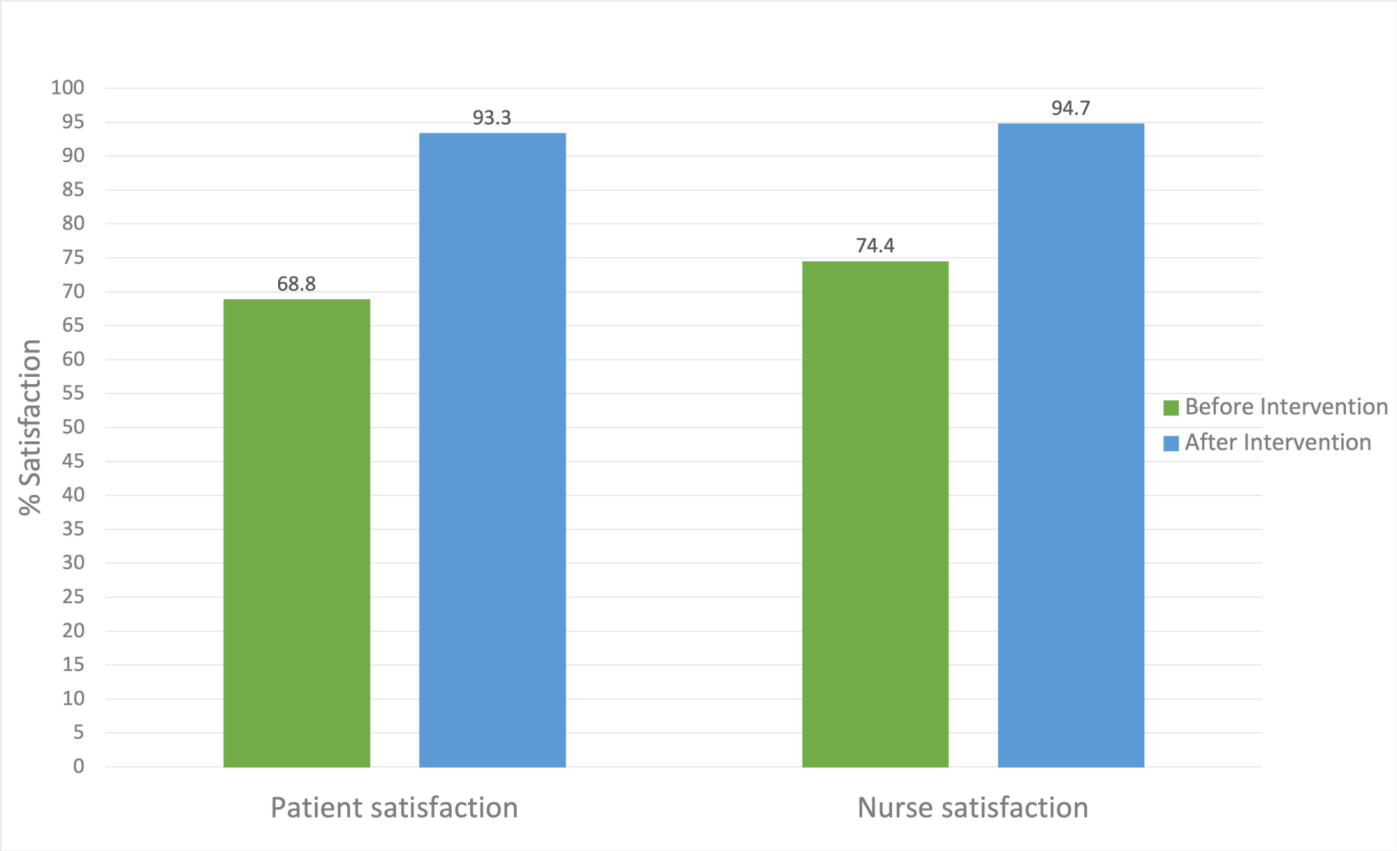
Satisfaction rates before and after the intervention

During the next six weeks, intervention in the form of visual handouts was implemented. During this period, a total of 40 patients and 40 nurses were included in the study. The results after the intervention revealed that 93.3% of patients were satisfied with the patient-provider communication, and 94.7% of nurses were satisfied with the nurse-provider communication (Figure 2).

At the end of the project, the Hospital Consumer Assessment of Healthcare Providers and Systems (HCAHPS) displayed an improvement in physician communication of 84.4%, up from 75% a year before.

## Discussion

Good physician-patient communication has the potential to help regulate patients’ emotions for the better, facilitate comprehension of medical information, and allow for better identification of patients’ needs, perceptions, and expectations [4,7-8]. Patients who report good communication with their doctors are more likely to be satisfied with their care [1,9]. Hence, patient satisfaction is one of the most recognized and widely used methods for assessing the effectiveness of physician-patient communication.

Multiple studies have been conducted on physician-patient communication, patient satisfaction, and patient outcomes. Research evidence suggests that patients want more involvement in their care [10,11]. Furthermore, dissatisfied patients have been shown to disenroll from health plans, engage in “doctor-shopping”, and they tend to be nonadherent to medical recommendations [12-15]. Therefore, good patient satisfaction is essential for the overall care of patients. A study by Marca-Frances et al. has reported that patients actively ask nurses for information and nurses would often be the ones receiving questions regarding their doubts [16]. Another study by von Knorring et al. has reported that improved teamwork between nurses and physicians was noticed by patients, which in turn has consequences for patient well-being [17-18]. These and multiple other studies suggest that improved physician-nurse communication leads to better outcomes for patients.

In our study, we used visual aids to assess and improve both physician-patient and physician-nurse communication. There was a notable improvement in both patients’ and nurses’ satisfaction rates. Visual aids that inform patients and nurses about the daily plan and their treatment team not only helped them understand their disease processes and further plans better but also promoted a better understanding of the roles of various physicians involved in their care. This, in turn, led to a better understanding of their diseases and overall care satisfaction. Since multiple studies have shown a correlation between patient outcomes and patient as well as nurse satisfaction rates, we hope to improve patient outcomes with this simple yet effective tool.

Limitations of our study include small sample size, the single-center nature of the study, and the short study period. Also, we could not factor in possible confounders. In the future, our steps would include disseminating visual aid tools across the entire patient population, mandating that daily medical care plan updates be handed out to the patients, and using electronic medical records to automatically generate visual aids whenever patients are admitted, to improve and help patient participation in decision-making right from the time of admission.

## Conclusions

Effective physician-patient and physician-nurse communications have been widely recognized as an integral part of healthcare delivery. Better communication has been proven to improve patient outcomes. There has been an increasing focus on improving communication in the healthcare system. Using visual aids and giving updated plans of care daily to the patients and their nurses can help improve communication significantly. These techniques should be adopted as widely as possible to improve patient outcomes.

## References

[1] Ha JF, Longnecker N (2010). Doctor-patient communication: a review. Ochsner J.

[2] Duffy FD, Gordon GH, Whelan G (2004). Assessing competence in communication and interpersonal skills: the Kalamazoo II report. Acad Med.

[3] Brinkman WB, Geraghty SR, Lanphear BP, Khoury JC, Gonzalez del Rey JA, Dewitt TG, Britto MT (2007). Effect of multisource feedback on resident communication skills and professionalism: a randomized controlled trial. Arch Pediatr Adolesc Med.

[4] Arora NK (2003). Interacting with cancer patients: the significance of physicians’ communication behavior. Soc Sci Med.

[5] Stewart MA (1995). Effective physician-patient communication and health outcomes: a review. CMAJ.

[6] Tang CJ, Chan SW, Zhou WT, Liaw SY (2013). Collaboration between hospital physicians and nurses: an integrated literature review. Int Nurs Rev.

[7] Platt FW, Keating KN (2007). Differences in physician and patient perceptions of uncomplicated UTI symptom severity: understanding the communication gap. Int J Clin Pract.

[8] Brédart A, Bouleuc C, Dolbeault S (2005). Doctor-patient communication and satisfaction with care in oncology. Curr Opin Oncol.

[9] Chandra S, Mohammadnezhad M, Ward P (2018). Trust and communication in a doctor-patient relationship: a literature review. J Healthc Commun.

[10] Vertinsky IB, Thompson WA, Uyeno D (1974). Measuring consumer desire for participation in clinical decision making. Health Serv Res.

[11] Haug MR, Lavin B (1979). Public challenge of physician authority. Med Care.

[12] Davies AR, Ware JE Jr, Brook RH, Peterson JR, Newhouse JP (1986). Consumer acceptance of prepaid and fee-for-service medical care: results from a randomized controlled trial. Health Serv Res.

[13] Kasteler J, Kane RL, Olsen DM, Thetford C (1976). Issues underlying prevalence of "doctor-shopping" behavior. J Health Soc Behav.

[14] Roter DL (1977). Patient participation in the patient-provider interaction: the effects of patient question asking on the quality of interaction, satisfaction and compliance. Health Educ Monogr.

[15] Francis V, Korsch BM, Morris MJ (1969). Gaps in doctor-patient communication. Patients’ response to medical advice. N Engl J Med.

[16] Marca-Frances G, Frigola-Reig J, Menéndez-Signorini JA, Compte-Pujol M, Massana-Morera E (2020). Defining patient communication needs during hospitalization to improve patient experience and health literacy. BMC Health Serv Res.

[17] von Knorring M, Griffiths P, Ball J, Runesdotter S, Lindqvist R (2020). Patient experience of communication consistency amongst staff is related to nurse-physician teamwork in hospitals. Nurs Open.

[18] Lyubovnikova J, West MA, Dawson JF, Carter MR (2015). 24-Karat or fool’s gold? consequences of real team and co-acting group membership in healthcare organizations. Eur J Work Organ Psychol.

